# NCEAS: Promoting Creative Collaborations

**DOI:** 10.1371/journal.pbio.0020072

**Published:** 2004-03-16

**Authors:** O. J Reichman

## Abstract

NCEAS -- an ecological synthesis center -- is changing the way ecological research is conducted by fostering new forms of collaboration and interdisciplinary research

A substantial portion of research occurs in places where scholars congregate—in campus laboratories, in libraries, or in large specialized facilities, such as oceanographic ships, astronomical observatories, or accelerators. Under any of these circumstances, researchers can interact easily, exchanging ideas and information.

Ecological research, especially the large component occurring in the field, takes place in disparate locations all over the world—in the air, on the surface, and below the oceans, lakes, and crust of the planet. Although there are many biological field stations where scientists and students gather, much of the research in ecology takes place in isolation. In addition to being highly dispersed geographically, ecology encompasses a disparate range of disciplines at scales from molecules to the globe, making the exchange of ideas and information even more difficult.

Recognizing the benefits of interaction and collaboration, the ecological community began considering a synthesis center where researchers from many fields could meet to address important ecological questions. Several organizations held discussions about the nature of such a center, which culminated in two workshops hosted by the National Science Foundation (NSF) in the early 1990s, to set the scope. In 1994, NSF conducted an open competition for a synthesis center, eventually granting the University of California, Santa Barbara, the award for the National Center for Ecological Analysis and Synthesis (NCEAS). In addition to funding from NSF, the center is supported by the University of California system and its Santa Barbara campus and by several foundations.

The center employs various types of research approaches. A primary approach is through Working Groups (Figure 1) where scientists come to NCEAS to concentrate on specific issues requiring synthesis of ideas, in-depth analysis of data, development of models, and preparation of results. The groups generally visit NCEAS two to four times over two years and stay for three to ten days at a time. Working Group topics range from microbial diversity to global change and have included projects in sociology, economics, and computer science. The Center hosts about 100 meetings a year, involving hundreds of participants.

**Figure 1:**
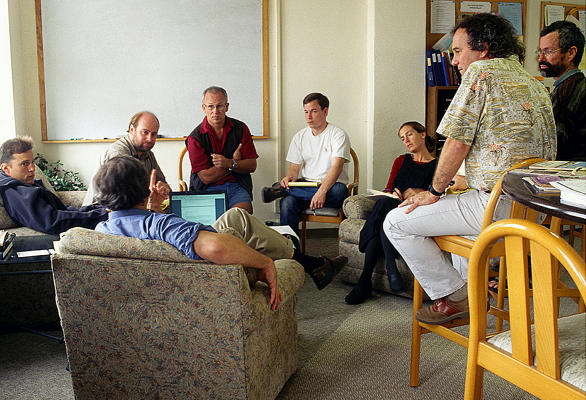
A Working Group at NCEAS on Diseases in Natural Populations (Photo used by permission from NCEAS.)

NCEAS also supports up to six visiting Center Fellows (sabbatical visitors) each year. These scientists often integrate their own research into a broader context or synthesize what is known about certain areas in ecology. Concurrently, the Center houses 15–18 Postdoctoral Associates for one to three years each. These postdoctoral positions are distinctive in that there are no permanent mentors for the larval scientists—rather, they interact with the other Associates, the resident Fellows, and the hundreds of individuals who annually visit the Center as part of Working Groups.

The Center also conducts training activities, including a distributed graduate seminar program. In this approach, graduate students around the world become involved in seminars on specific topics using data from their region (e.g., the relationship between productivity and diversity) and then representatives from each of the seminars are brought to NCEAS for a grand synthesis.

As would be expected for a discipline as broad as ecology, the participants at NCEAS are extremely diverse. Over 3,000 individuals have visited NCEAS in just over eight years, representing 43 countries and 49 states in the United States. They come from over 800 institutions, many non-biology departments, and 397 non-academic organizations (e.g., agencies and companies). An interesting measure of their breadth is that participants belong to more than 180 professional societies.

Proposals are solicited twice a year and reviewed by a Science Advisory Board. The Board looks for topics that would benefit from synthesis and analysis and that would make significant contributions to our understanding of ecological relationships. While many proposals pertain to core ecological questions, others deal with economics or sociology (e.g., how metaphors affect the way we conduct research). Approximately 40% of the projects have some applied component, many influencing resource management practices and conservation policies.

Because the Center is based on the use of existing data, access to highly dispersed and profoundly heterogeneous ecological information is essential, but also very difficult. Recognizing the need for open access to a wide variety of data—versus project-specific data solutions—NCEAS and several collaborators (see http://www.ecoinformatics.org) have embarked on a major research program in developing tools to characterize data and make them available in standardized formats. The initial research effort, called the Knowledge Network for BioComplexity (KNB) is yielding tools to generate metadata (precise information about the data) and to make all the data available. The current research thrust, called Science Environment for Ecological Knowledge, will expand the capabilities of KNB by employing grid technology (in particular, EcoGrid, a network of networks), semantic mediation, knowledge representation, and workflow models for analysis and synthesis.

The Center has supported almost 200 projects, the results of which are published in top scientific journals (see project results at http://www.nceas.ucsb.edu). Furthermore, some of the projects have had direct influence on conservation and resource management. For example, scientists at NCEAS developed theories for the design of marine reserves that were soon thereafter applied to the placement of reserves directly off the coast of Santa Barbara.

In addition to scientific results, NCEAS is changing the way we conduct ecological research through novel means of encouraging productive collaborations. Sociologists studying the NCEAS model of collaboration have identified several important factors in its success. These include a distant, neutral location facilitating periodic, highly focused opportunities to concentrate on the issues under consideration; logistic support that lowers the activation energy required to develop collaborations; and the proximity of scientists from many disciplines having the opportunity to interact in ways otherwise not possible.

Many significant contributions to our understanding of the patterns and processes of the natural world have emerged from NCEAS research activities. In addition, the Center is fostering new forms of collaboration and interdisciplinary research by providing a place where scientists from many disciplines can productively interact and by working to make eclectic ecological data available to many users. This is an extremely simple model for the scientific enterprise—but one not captured in most existing institutional and organizational structures. As recognition of the success of NCEAS spreads, other institutions are attempting to incorporate some of the traits of the Center into their operations, and new centers are being proposed. For example, the NSF is in the midst of a review of proposals for an evolution synthesis center.

It is clear that the complexity of ecological systems, as well as the importance of understanding and maintaining them, requires information and knowledge from many disciplines. This is true even at a time when disciplines are becoming more specialized and scientists have less time to concentrate on broader issues. By facilitating interactions among many scholars and practitioners, NCEAS provides both time when and a place where far-reaching topics can be addressed.

**Figure:**
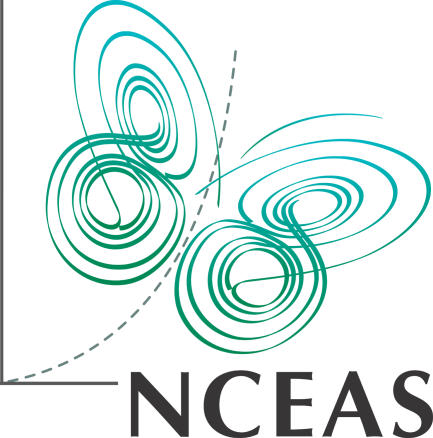


## Abbreviations

KNB: Knowledge Network for BioComplexity
NCEAS: National Center for Ecological Analysis and Synthesis
NSF: National Science Foundation
